# Polyostotic Fibrous Dysplasia Mimicking Osseous Metastases

**DOI:** 10.7759/cureus.3884

**Published:** 2019-01-14

**Authors:** Luwei Tao, Jingxin Sun, Akriti Jain, Jie Ouyang, Daniel Tambunan

**Affiliations:** 1 Internal Medicine, Florida Hospital, Orlando, USA; 2 Pathology, Florida Hospital, Orlando, USA

**Keywords:** fibrous dysplasia, polyostotic, neoplasm

## Abstract

Fibrous dysplasia (FD) is a benign bone disorder, in which normal bone structure is replaced by fibrous connective tissue. Polyostotic FD is also related to McCune-Albright syndrome with possible endocrine disorder and Cafe-au-lait macules. Although FD commonly presents as craniofacial bone abnormality, atypical presentation can be misleading and pose a difficulty in clinical diagnosis. Here we report a case of polyostotic FD, who presented as an accidental finding of multiple spinal osseous lesions, leading to clinic workup for metastatic cancer.

## Introduction

Fibrous dysplasia (FD) is a rare non-neoplastic bone disorder featured by replacement of bone with fibrous connective tissue, which causes thinning of cortical bone, deformity, chronic pain and nerve compression [1]. The pathogenesis of FD involves mutation of GNAS1 gene and constitutive activation of G proteins, leading to proliferation of mesenchymal cells [2]. FD accounts for 5-7% of all benign bone lesions [1]. Depending on the number of bones involved, FD can be monostotic or polyostotic. While monostotic lesions usually arrest with onset of puberty, polyostotic lesions continue to progress through adulthood [1, 2]. The classic triad of McCune-Albright syndrome includes polyostotic FD (PFD), Cafe-au-lait macules and endocrinopathy. First symptoms of FD usually appear between 5 and 20 years of age and craniofacial skeleton abnormalities, including bone deformity and cranial nerve impingement, are common initial presentations [1, 3]. Here we report a rare case of PFD, which initially presented with accidental findings of multiple spinal bone lesions, mimicking metastatic cancer.

## Case presentation

**Figure 1:**
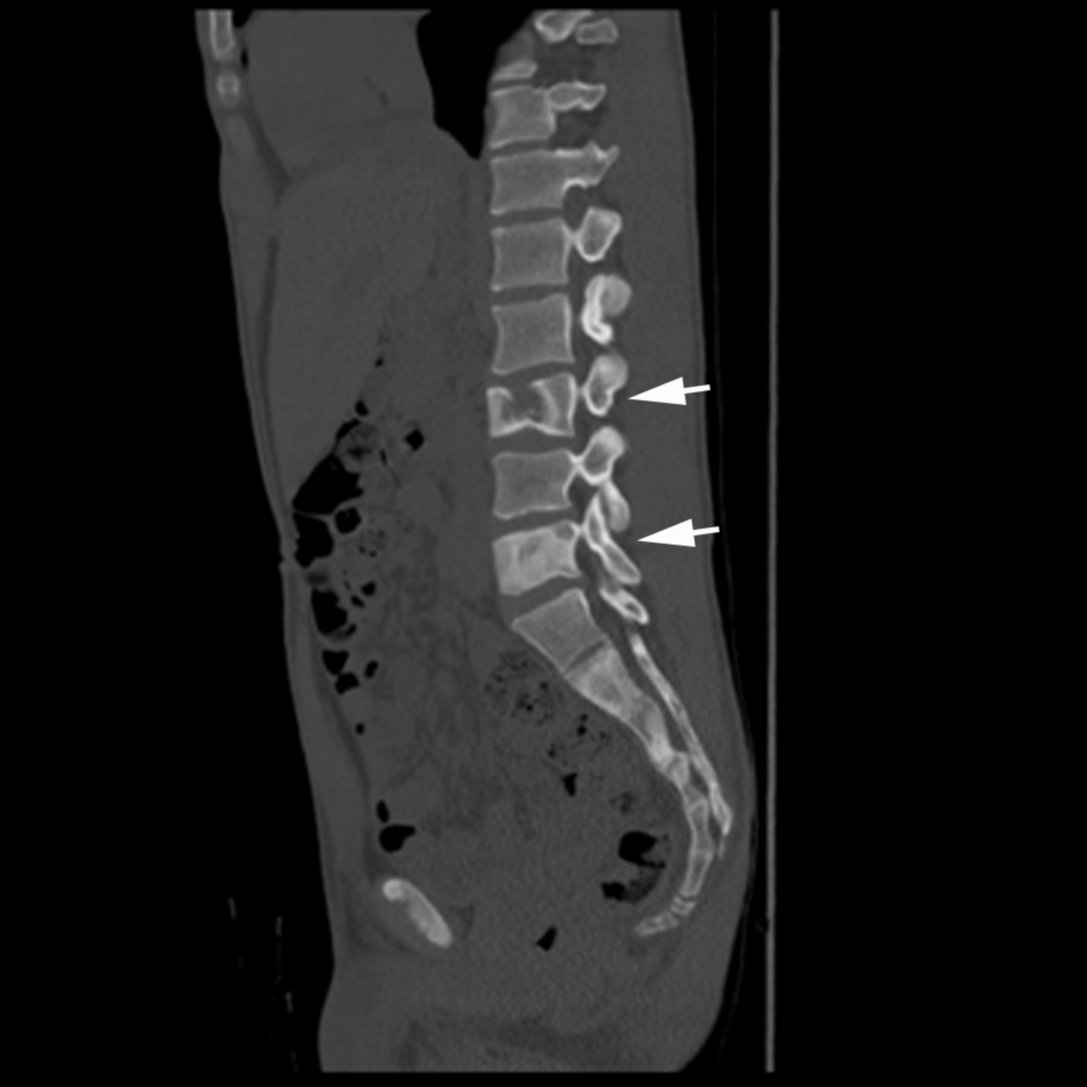
Admission computed tomography (CT) abdomen showed multiple osseous lesions with both osteosclerotic and osteolytic features as indicated by arrows.

**Figure 2:**
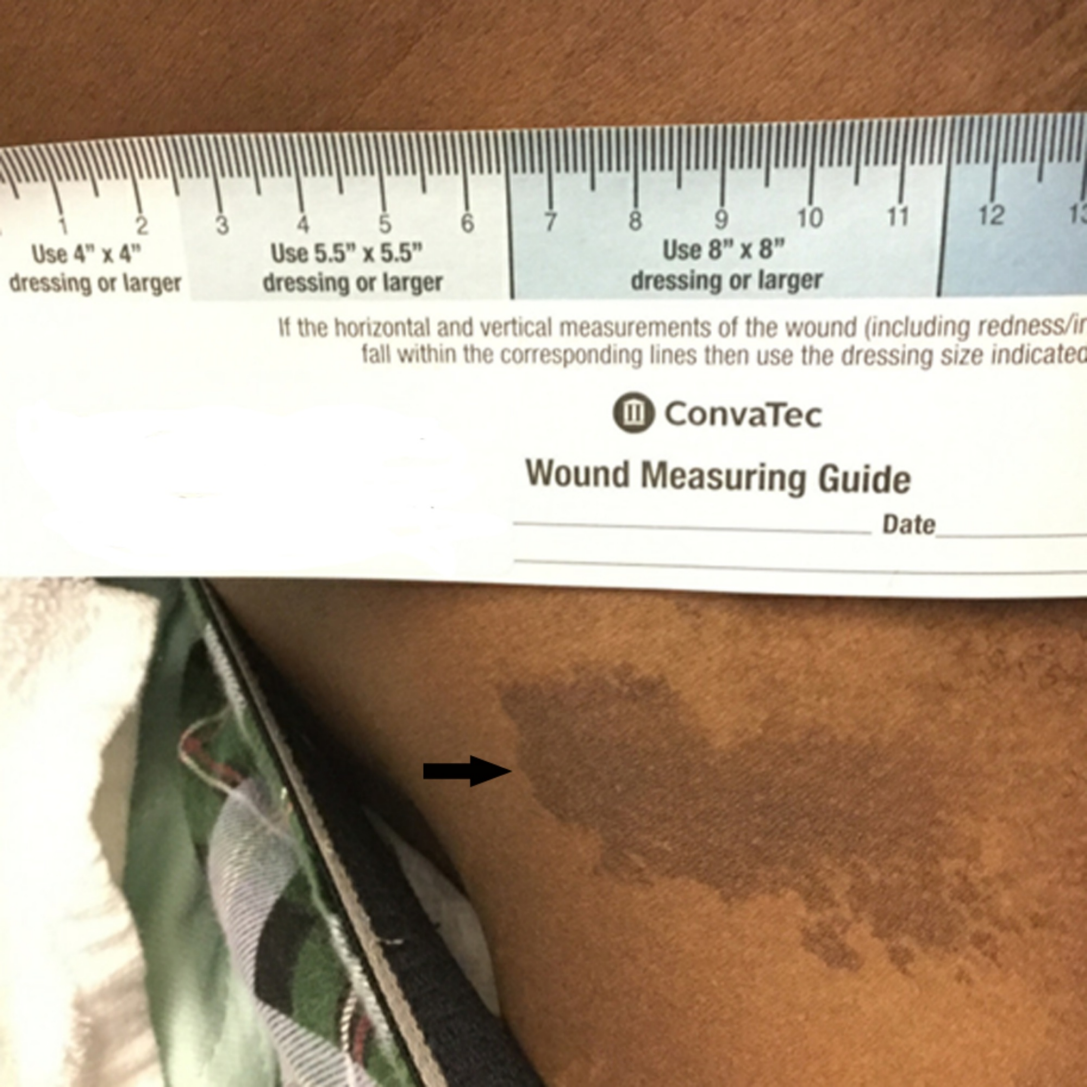
Cafe-au-lait macule on patient's right thigh with “coast of Maine” feature.

A 19-year-old male presented to our emergency room with one day history of left flank pain and dysuria. He endorsed mild chronic mild low back pain, which did not impair his daily activities and which he attributed to a previous car accident. During initial workup for hematuria, abdominal computed tomography (CT) scan revealed diffuse lumbar spinal osseous lesions with both osteoblastic and osteolytic features (Figure 1). The patient was therefore admitted to internal medicine service for workup of potential metastatic malignancy. His grandmother had cervical cancer; otherwise his family history was unremarkable. Physical examination showed mild tenderness to palpation on thoracic and lumbar spine. His right orbital rim was slightly more prominent than left side and there was a dark skin mark on right front thigh along L1-2 distribution (Figure 2).

During the hospital stay, the patient’s laboratory tests were significant only for bacteriuria and vitamin D deficiency with a normal complete blood count/comprehensive metabolic panel, including alkaline phosphatase. Serum protein electrophoresis was negative for any M protein. Tumor markers including CEA and PSA were negative.

**Figure 3:**
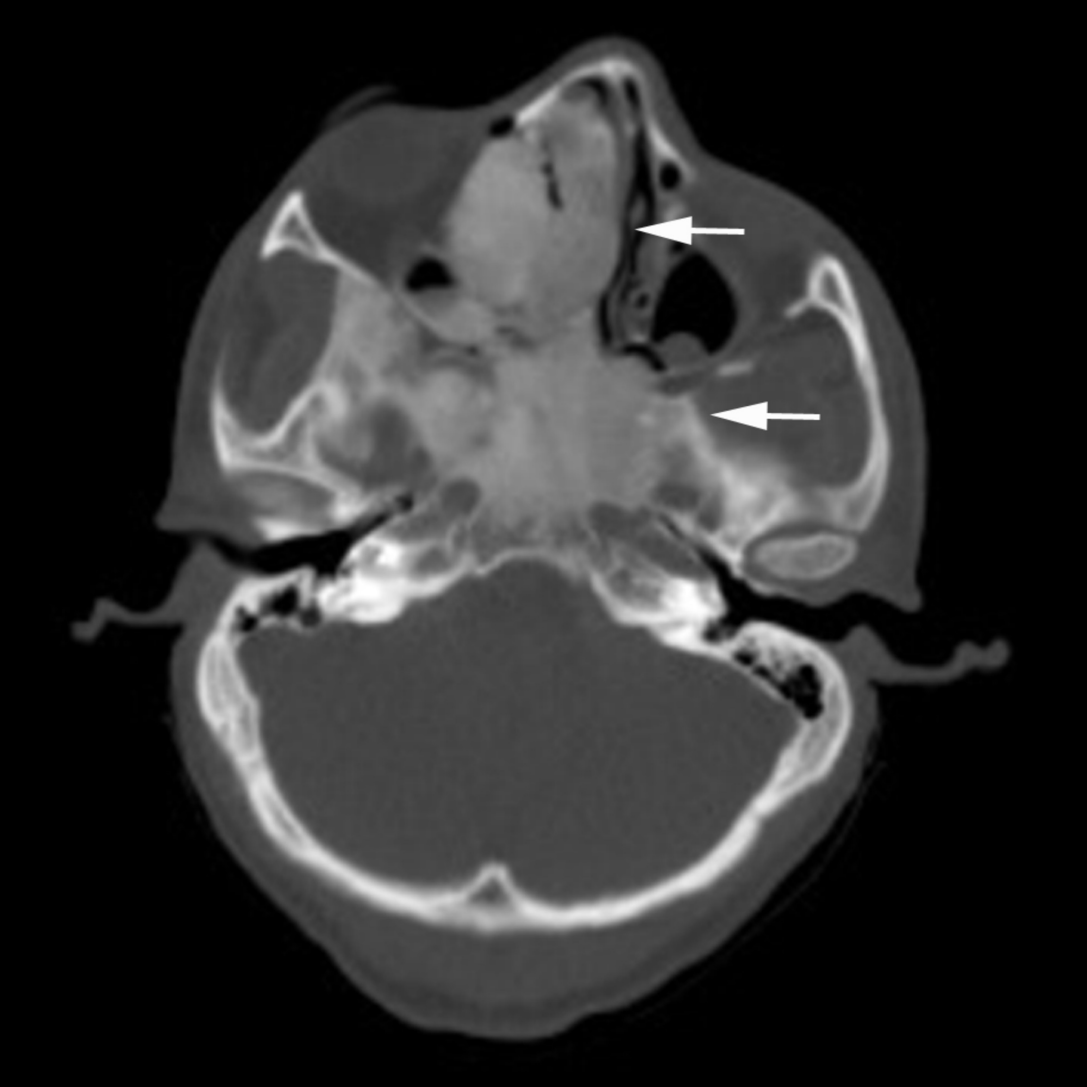
Single photo emission computed tomography (SPECT) head showed typical para-orbital and para-nasal ground-glass lesions as indicated by arrows.

**Figure 4:**
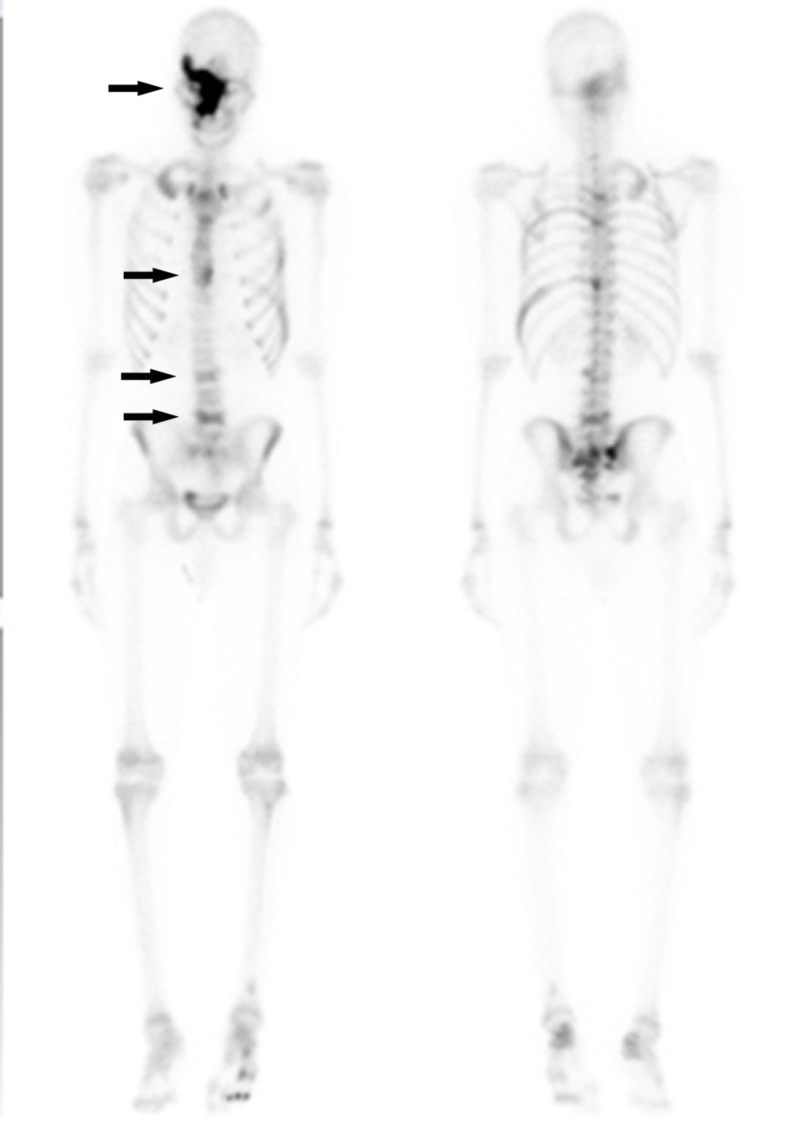
Nuclear bone scan showed all lesions with increased nuclear signal uptake as indicated by arrows.

**Figure 5:**
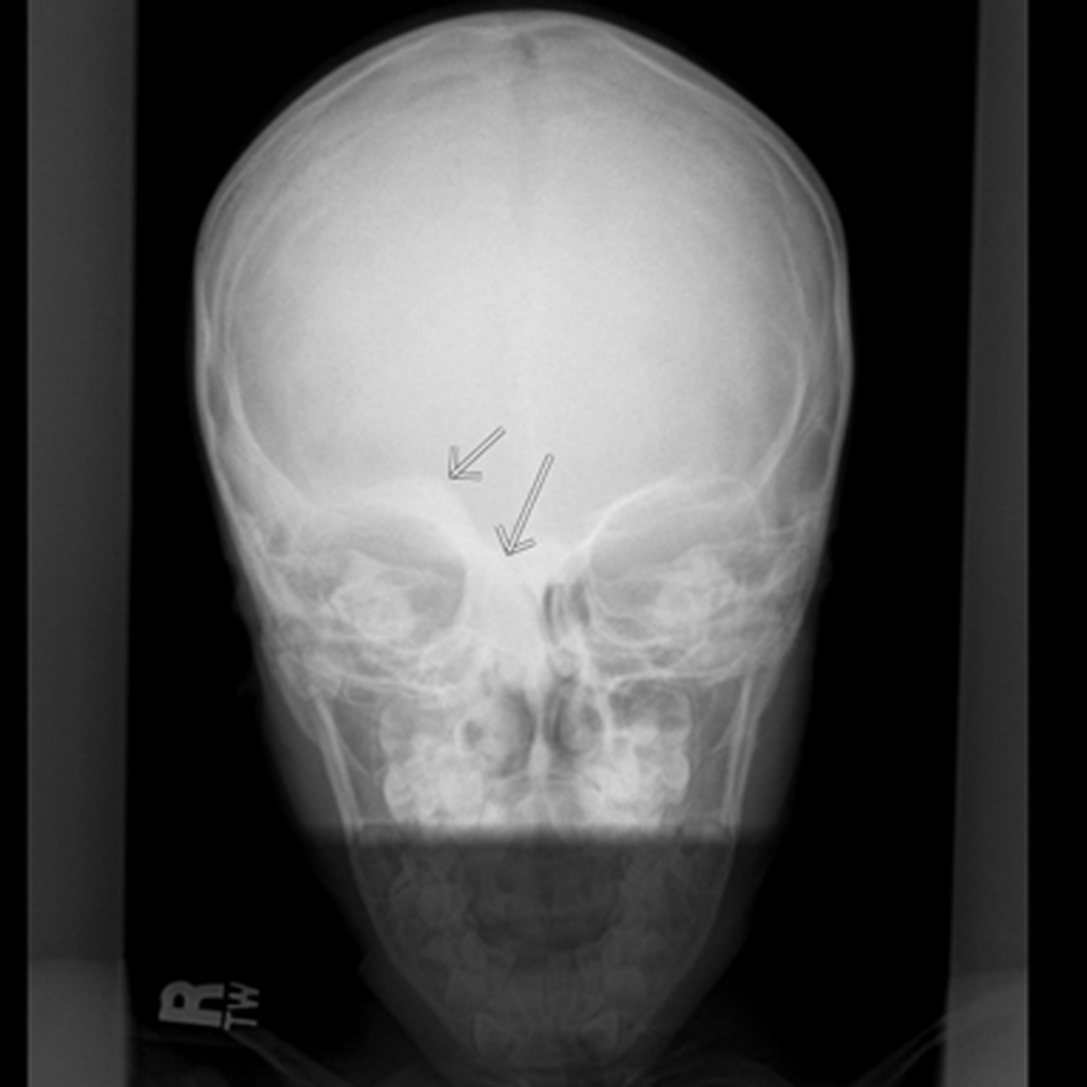
Skull X-ray of the same patient at five years of age with sclerotic lesion on right orbital rim as indicated by arrows.

**Figure 6:**
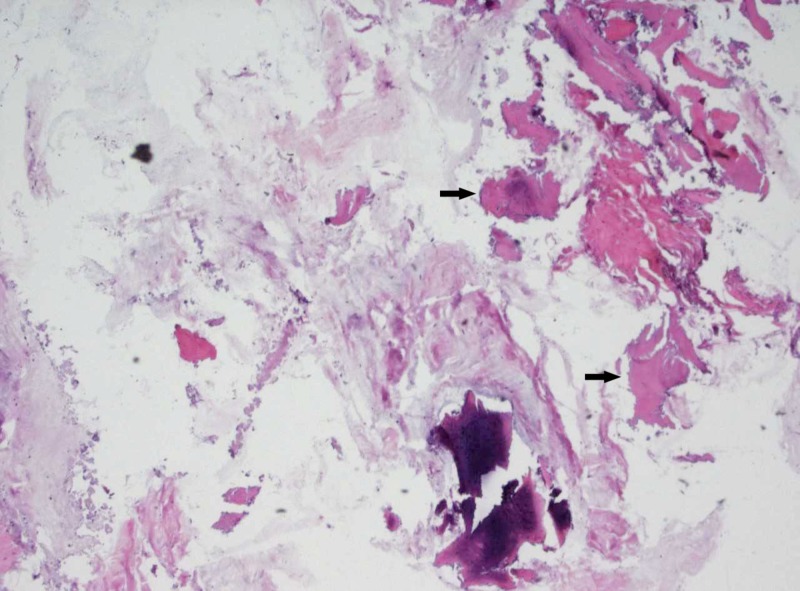
Bone biopsy showed fragment of cartilage and bone as indicated by arrows. No malignancy was identified.

Chest, abdominal and pelvic CT scan with contrast was carried out, which showed additional similar thoracic spinal lesions. No primary tumor lesion was identified, except a small 4 mm non-specific pulmonary nodule. Single photo emission computed tomography (SPECT) and whole body nuclear scan were carried out for further characterization of patient multiple bone lesions. SPECT showed ground-glass sclerotic lesions on maxillofacial region and right calvarium, which is typical for fibrous dysplasia (Figure 3). All bony lesions are associated with increased nuclear signal uptake (Figure 4). Retrospectively, the patient reported to have a small lump on right forehead at the age of five, for which he had an X-ray. We were able to retrieve the original image and found that the same sclerotic lesion on right orbital rim had remained stable in the past 14 years, further suggesting FD, a benign disease process (Figure 5). Finally, a bone biopsy was performed, which showed fragment of cartilage and bone without malignancy (Figure 6).

Based on clinical presentation and imaging, this patient was considered to have PFD with possible McCune-Albright syndrome, although there was no sign of endocrinopathy yet. The patient was discharged with calcium and vitamin D supplements. Intermittent low dose acetaminophen or non-steroid anti-inflammatory drugs (NSAIDs) were recommended for symptomatic relief of mild lower back pain. Consultation for surveillance of future endocrine disorder was also provided before the patient was discharged.

## Discussion

FD is a rare skeletal disorder, which is often encountered in adolescents and young adults during the investigation for an unrelated cause. Clinical manifestation of FD varies greatly, depending on the affected skeleton [4, 5]. Painless bony enlargement of part of the face is the usual presentation in the first decade of patient's life. If bone structures of skull base or posterior orbit are involved, the patient can develop vision loss, hearing impairment or nerve palsy [5, 6]. It is important to recognize that PFD can also present with multiple bone sclerotic/lytic lesions, especially in young patients. Early clinical suspicion can accelerate diagnosis and minimize patient’s anxiety. Differential diagnosis includes other common etiologies, such as multiple myeloma, prostate cancer, myeloproliferative disorders and infection. CT is the choice of initial evaluation [6]. Typical lesions have “ground-glass appearance” (Figure 3). Magnetic resonance imaging (MRI) can be used to visualize nearby structural compression. And positron emission tomography (PET)-CT, usually as a part of metastasis workup, shows high nuclear signal enrichment [7]. Bone biopsy is often necessary and confirmatory, especially when there is a concern for malignancy as in our case [7, 8].

PFD can be associated with skin cafe-au-lait pigmentation, endocrine abnormality, and this classic triad is known as McCune-Albright syndrome (MAS), which is caused by genetic mutation of GNAS gene. In contrast to cafe-au-lait spots with smooth borders in other disorders, the borders of cafe-au-lait spots in MAS are often irregular and described as coast of Maine (Figure 2). Girls with MAS may show precocious puberty caused by excessive estrogen. Less commonly, boys with MAS may also have early puberty, which is absent in this reported case. Endocrine problems may also affect other organs, including thyroid gland, pituitary gland and, rarely, adrenal gland. Patients need to be advised to have regular check up for potential endocrine disorders.

The treatment of polyostotic FD focuses on symptomatic control. Pain is common among FD patients. Some patients require NSAIDS for pain control with or without narcotics. The use of bisphosphonates for pain control has been reported. In small non-randomized trials, intravenous infusion of pamidronate is associated with reduced bone pain, decreased bone turnover, reduced fracture and increased bone density [9, 10]. However, it is controversial whether bisphosphonates can change progression of the disease process [11, 12]. For patients who failed the treatment with bisphosphonates, the use of denosumab has also been reported [13]. Surgical intervention is usually necessary for lesions which are cosmetically significant or symptomatic [6]. For patients with clinical impairment of optic nerve, urgent surgical decompression is indicated [14]. However, for asymptomatic patients with radiographical involvement of optic nerve, watchful waiting showed superior clinical outcome and prophylactic surgical decompression is not indicated [14].

## Conclusions

Fibrous dysplasia is a rare benign bone disorder featured with replacement of bone with fibrous connective tissue, which can mimic metastatic bone disease. It is important to recognize that PFD can also present with multiple osseous lesions, especially in young patients. Early clinic suspicion can accelerate diagnosis, minimize unnecessary workup, cost and length of stay.
